# Outcomes of patients undergoing anatomical total shoulder arthroplasty with augmented glenoid components – a systematic review

**DOI:** 10.1177/17585732231192991

**Published:** 2023-08-07

**Authors:** Carlos Prada, Omar A Al-Mohrej, Salwa Siddiqui, Moin Khan

**Affiliations:** 1 Division of Orthopaedic Surgery, Department of Surgery, 3710 McMaster University, Hamilton, Ontario, Canada; 2 Department of Health Research Methods, Evidence, and Impact, 3710 McMaster University, Hamilton, Ontario, Canada; 3 Section of Orthopedic Surgery, Department of Surgery, 430300 King Abdullah Bin Abdulaziz University Hospital, Princess Nourah Bint Abdul Rahman University, Riyadh, Saudi Arabia; 4 School of Nursing, 3710 McMaster University, Hamilton, Ontario, Canada

**Keywords:** augmented glenoids, anatomic total shoulder arthroplasty, shoulder arthroplasty, glenoid components

## Abstract

**Background:**

Glenoid loosening is an issue in anatomic total shoulder arthroplasty (a-TSA). This has been attributed to abnormal glenoid anatomy, common among these patients. Different alternatives have been proposed to tackle glenoid bone loss and restore joint alignment with augmented glenoid implants being increasingly used to deal with this problem. This systematic review aims to evaluate the clinical and radiological outcomes of patients undergoing augmented glenoid a-TSAs. Our hypothesis was that augmented glenoid components will lead to good patient outcomes with a low incidence of complications and revision procedures.

**Methods:**

MEDLINE, EMBASE, CENTRAL and CINHAL were searched from inception to February 2022 for information pertaining to outcomes of patients undergoing a-TSA with augmented glenoid implants.

**Results:**

Eighteen studies reported on outcomes of 814 a-TSA (800 participants) with a mean follow-up of 3.7 years. Most studies (67%) were Type IV level of evidence. Almost 70% of participants underwent an a-TSA secondary to primary glenohumeral osteoarthritis. Most glenoids were type B2 (73%). Augmented glenoids material was mostly all-polyethylene (81%) with full wedge (45%) and stepped components (38%) designs being the most common. Most studies reported good clinical outcomes. 17 patients (4%) underwent a revision surgery.

**Conclusions:**

Our review found that patients undergoing a-TSA with augmented glenoid components report good outcomes at short-to-mid-term follow-up. Further research is warranted to determine if such outcomes remain similar in long term.

**Level of evidence:**

Level III, Systematic Review of Therapeutic Studies.

## Introduction

Shoulder arthroplasty has evolved significantly since Neer developed what is considered the first of the ‘modern era’ implants in 1955.^1^ During the last few decades, there has been an increase in the number of total shoulder arthroplasties performed worldwide and is expected to continue on the rise.^2,3,4,5^

Anatomic total shoulder arthroplasty (a-TSA) is the preferred option when dealing with shoulder arthritic conditions in the presence of a functional rotator cuff. Here, unlike what occurs with reverse shoulder arthroplasty (RSA), the shoulder surfaces are replaced by a glenoid component and a humeral component that follow the natural joint configuration. However, often patients undergoing a-TSA have abnormal glenoid anatomy which poses a challenge for surgeons, so it is not surprising that most research has been around glenoid deformities and how to address them properly during shoulder arthroplasty.

Historically, glenoid components of a-TSAs accounted for most implant failures, with glenoid loosening being pointed out as one of the leading causes of revision. Frequently, this has been attributed to the inability to achieve correct glenoid component positioning during the surgical procedure due to glenoid bone loss and retroversion. Many strategies have been described to tackle this problem: (a) eccentric reaming; (b) bone grafting; (c) RSA; and (d) augmented glenoid (AG) components have all been proposed as possible solutions, but there is a lack of high-quality evidence to guide surgeons in the best treatment option when dealing with axial misalignments above 15°.

AG implants are an attractive option because they preserve bone stock (compared with eccentric reaming), decrease joint line medialization, and are biomechanically stronger than constructs using bone grafting.^6^ However, initial results were discouraging with early designs showing instability and suboptimal correction.^7,8^

Given the lack of robust evidence on this topic, it is still a matter of debate amongst surgeons about what is the best option to face patients undergoing a-TSA with glenoid axial malalignments above 15°. There are some concerns about using an RSA as the first option owing to the potential need for future revision procedures and its challenges. On the other hand, eccentric reaming has been proposed, but excessive reaming increases the risk of glenoid component medialization and interface with a softer bone elevating the risk of peg perforations posing the glenoid component at risk for subsequent loosening or instability.

Two previous systematic reviews have studied the outcomes of a-TSA in patients with posterior glenoid wear (Walch type B2 glenoids),^9,10^ but, to our knowledge, no evidence-synthesis has been conducted specifically on AG implants and their outcomes. Thus, the purpose of this systematic review was to evaluate current evidence with respect to clinical and radiological outcomes of patients undergoing AG a-TSAs. Our hypothesis was that AG components will lead to good patient outcomes with a low incidence of complications and revision procedures.

## Methods

The study was conducted according to the Cochrane Handbook for Systematic Reviews of Interventions Version 6.0^11^ and reported as per the Preferred Reporting Items for Systematic Reviews and Meta-Analyses (PRISMA) guidelines.^12^

### Search strategy

Excerpta Medica Database (EMBASE), Medical Literature Analysis and Retrieval System Online (MEDLINE/PubMed), Cochrane Central Register of Controlled Trials (CENTRAL), and Cumulative Index to Nursing and Allied Health Literature (CINAHL) database were used. They were searched from the date of establishment to 22 February 2022.

The search strategy, adapted to each database, included terms representing the use of AG implants in a-TSA and its outcomes (Appendix 1 in the Supplemental Material). MeSH and EMTREE terms were used, along with free text, in several combinations to improve the search sensitivity. Reviewers conducted a manual review of references from included studies to identify any studies that may have been missed in the initial search strategy.

### Study selection

Study selection was performed by two independent authors (CP and OAM) using Covidence (Covidence systematic review software, Veritas Health Innovation, Melbourne, Australia). The screening of study headings, abstracts and full text was done in duplicate. A resolve-by-consensus strategy was utilized for all discrepancies. If a consensus could not be reached, a senior reviewer (MK) was consulted. An unweighted kappa score was used to measure the level of agreement.

### Eligibility criteria

All studies assessing any type of outcome of AG implants in a-TSA were included. Published studies that reported at least one outcome related to AG implants were eligible for inclusion. The exclusion criteria were: (a) case-series (less than five patients); (2) review articles and conference abstracts; and (3) language other than English. In addition, studies which reported on the outcomes of patients included in previous studies were excluded.

### Data collection

A collaborative pre-defined data abstraction web-based spreadsheet (Google Sheets 2021; Google LLC, California, USA) was used for data collection. Data extraction was not done in duplicate but to ensure accuracy, the results were audited by both reviewers and reviewed by the senior author. Extracted data included information on basic study characteristics (authors, publication year, country, study type and level of evidence), demographic data (age, sex, sample size, and indication/diagnosis), AG implants characteristics (material and type of augment), outcomes, and complications.

### Risk of bias and quality assessment

The methodological index for non-randomized studies (MINORS) was used by the two reviewers to assess the risk of bias in included studies.^13^ The MINORS scale assigns a score of 0, 1, or 2 for a list of 8 and 12 domains in non-comparative, and comparative studies.

MINORS quality was categorized a priori for non-comparative studies as high quality of evidence (score 13–16); fair quality of evidence (score 9–12); and poor quality of evidence (score 0–8). For comparative studies, high quality of evidence (score 17–24), fair quality of evidence (score 13–16), and poor quality of evidence (score 0–12).

### Statistical analysis

Descriptive statistics were calculated to reflect the frequency and percentage of abstracted study data.

Using Cohen's Kappa (κ), inter-reviewer agreement at each stage of the screening process was calculated. An agreement was categorized a priori, as per Landis and Koch, with *k* of 0.81 to 1.0 for near-perfect agreement; *k* of 0.61 to 0.80 for substantial agreement; *k* of 0.41 to 0.60 for moderate agreement; and *k* of 0.21 to 0.40 as fair agreement. Interobserver agreement for methodologic quality assessment was calculated using the intraclass correlation coefficient (ICC); a value of ≥0.65 was considered adequate.^3^

## Results

### Study characteristics

**Figure 1.**
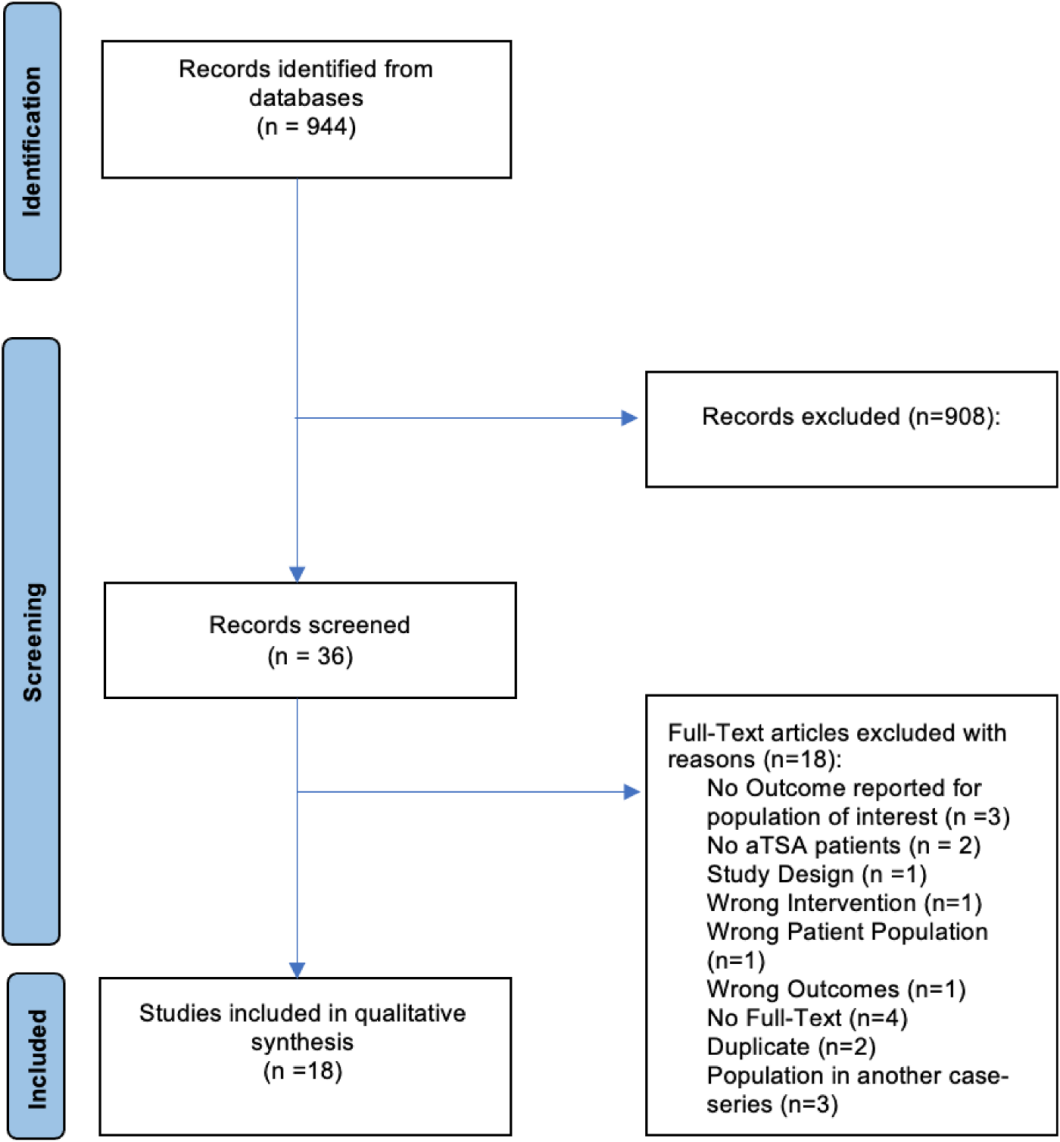
Preferred reporting items for systematic reviews and meta-analyses (PRISMA) flow diagram.

Of 944 studies retrieved using our search strategy, 36 studies underwent full-text review. Eighteen studies were included (Figure 1).^8,14–30^ The agreement for title and abstract screening was substantial (*k* = 0.65), while the agreement for full-text review was moderate (*k* = 0.43). There was a near-perfect agreement in the quality assessment scores using the MINORS criteria (ICC 0.91). The mean MINORS score was 9.7 ± 1.6 out of 16 for non-comparative studies and 14.6 ± 3.6 out of 24 for comparative studies.

This review includes a total of 800 patients (814 shoulders). Fourteen studies reported on patient's demographics (78%, *n* = 637 patients), 31% (197) were female. Of the 814 shoulders included, 70% (570) received AG components, 20% (166) received standard glenoid (SG) components in a matched-cohort, 6% (48) underwent asymmetric reaming followed by standard components, and 4% (30) were treated with either an angled keel or extra-thick glenoid components. The mean age of the participants was 70.2 years (range 34–85) although two studies grouped age with their matched cohorts. Most studies assessed outcomes on posterior AG components (16 studies, 94%). One study (6%) assessed outcomes on anterior AG components. Follow-up was reported in 14 studies (78%) with a mean of 3.7 years (range 0.1–19.1).

Studies were published between 2008 and 2022, with 11 (61%) being published within 5 years of the search. Out of the 18 included studies, one (6%) was of level II evidence (172 patients; 62 AG and 110 SG). Four studies (22%) were of level III evidence with 256 patients (163 AG and 93 SG). The remaining 12 studies (67%) were of level IV evidence, with a sample of 473 patients (395 AG and 48 SG after asymmetric reaming, 18 received angled keel glenoid components, and 12 received extra-thick glenoid components). Most studies were conducted in the United States (*n* = 15, 83%). Details regarding the study characteristics and patients’ demographics of included studies are found in Table 1.

**Table 1.** Study characteristics and methodological quality of included studies.

References	Study type	Study design	Level of evidence	Country	Sample size	Mean age (years)	Sex (% female)	Mean follow-up (months)	MINORS Score
Cronin et al.^14^	Prognostic	Case studies	IV	USA	42	63	12 (28.6%)	N/R	11/16
Wright et al.^15^	Therapeutic	Matched cohort retrospective	III	USA	74	PAG: 64.3 SG: 65	AG: 12 (32.4%) SG: 12 (32.4%)	24	12/24
Michael et al.^16^	Therapeutic	Matched cohort retrospective	III	USA	73	N/R	N/R	Cage 8° augment: 17.7 8° poly: 38.1	10/24
Sabesan et al.^17^	Anatomy study, imaging and computer modelling	N/R	N/R	USA	29	66.9	5 (17.2%)	NR	N/A
Ricchetti et al.^18^	Therapeutic	Prospective cohort design	II	USA	172	63	N/R	NR	19/24
Lenart et al.^19^	Therapeutic	Case-series	IV	USA	5	67.4	5 (100%)	33.2	10/16
Terrier et al.^20^	Basic science	Basic science	IV	Switzerland	9	62.8	5 (56%)	11.5	11/16
Sandow and Tu^21^	Therapeutic	Case-series	IV	Australia	141	*Range 42–85*	23 (34.8%)	48	9/16
Stephens et al.^22^	Therapeutic	Case-series	IV	USA	21	66	N/R	35	10/16
Ho et al.^23^	Therapeutic	Retrospective cohort	IV	USA	71	65	16 (22.5%)	28.8	10.5/16
Priddy et al.^24^	Therapeutic	Matched cohort retrospective	III	USA	74	64.1	AG: 11 (29.8%) SG: 11 (29.8%)	38.4	10/16
Ko et al.^25^	Therapeutic	Retrospective cohort	IV	USA	97	AR: 65.3 PAG: 67.4	AR: 12 (33.3%) PAG: 20 (40.8%)	N/R	17/24
Rice et al.^26^	Therapeutic	Retrospective cohort	IV	USA	13	66	N/R	60	15/24
Favorito et al.^27^	Therapeutic	Case-series	IV	USA	19	62	4 (21.1%)	36	10/16
Cil et al.^8^	Therapeutic	Case-series	IV	USA	38	65	12 (31.6%)	87.6	12/16
Das et al.^29^	Therapeutic	Retrospective cohort	IV	UK	10	59	5 (50%)	19	10/16
Grey et al.^30^	Therapeutic	Case Series	IV	USA	68	64.9	24 (35.2%)	49.9 ± 18.2	10/16
Kohan et al.^28^	Therapeutic	Retrospective cohort	III	USA	35	SG: 68 ± 8.6 AG: 70.0 ± 8.7	SG: 3 (16%) AG: 1 (6%)	104.4	17/24

PAG aTSA: posterior augmented glenoid with anatomic total shoulder arthroplasty; SG: standard group (atomic total shoulder arthroplasty without posterior glenoid wear); AG: augment group.

### Diagnosis and glenoid type

Twelve (67%) studies reported the diagnosis that led to the surgery in 473 cases. Most patients underwent an augmented a-TSA because of primary glenohumeral osteoarthritis (*n* = 452, 96%). Thirteen patients (3%) underwent an AG a-TSA as a revision procedure (eight aseptic loosenings or component instability and five failed hemiarthroplasties).

From the 570 shoulders (70%) that underwent an augmented a-TSA, 437 (77%) reported on the glenoid type according to Walch et al.’s classification. Most glenoids were type B2 (*n* = 321, 73%), followed by B3 glenoids (*n* = 85, 19%), and B1 and C type glenoids (*n* = 14) representing 3% each. Further details can be found in Table 2.

**Table 2.** Diagnosis and glenoid type.

References	Indications	Type of augment (shape)	Material of augment	Degrees of augmentation
Cronin et al.^14^	An augment was considered when with SG it was not possible to achieve: <10° retroversion, <10° superior inclination, >90% backside contact, <2 mm medial reaming, and <1 peg perforation	N/R	N/R	N/R
Wright et al.^15^	Glenoid osteoarthritis	N/R	N/R	N/R
Michael et al.^16^	N/R	8° cage, All-poly 8° + All-poly 16°	Mixed	8° and 16°
Sabesan et al.^17^	Glenohumeral osteoarthritis	Stepped and monoblock	Polyethylene (Global Steptech)	Augments of 3 mm (*n* = 7), 5 mm (*n* = 7) or 7 mm (*n* = 14)
Ricchetti et al.^18^	Advanced glenohumeral osteoarthritis	Full-wedge and monoblock	Polyethylene	N/R
Lenart et al.^19^	Anterior glenoid erosion (*n* = 2), malunited glenoid fracture (*n* = 1), non-united glenoid fracture and post-traumatic arthritis	Stepped and monoblock	Polyethylene	4 patients + 3, 1 patient + 5
Terrier et al.^20^	Primary glenohumeral osteoarthritis	Half-wedge and monoblock	Keeled PAG implants (Aqaelis Perform)	Posterior augments of 15°, 25°, or 35°
Sandow and Tu^21^	Walch B2 and type C glenoids	Half-wedge and monoblock	Metal	15° and 30°
Stephens et al.^22^	Glenohumeral osteoarthritis	Stepped and monoblock	Polyethylene	3 mm augment (*n* = 7) and 5 mm augment (*n* = 14)
Ho et al.^23^	Primary glenohumeral osteoarthritis	Stepped and monoblock	Polyethylene	3 mm augment (*n* = 28), 5 mm (*n* = 30), and 7 mm (*n* = 13)
Priddy et al.^24^	All primary TSAs were identified through a single institution's shoulder arthroplasty database	Full wedge and monoblock	Polyethylene peg glenoid (Equinoxe)	8° (*n* = 32), 12° (*n* = 1), and 16° (*n* = 4)
Ko et al.^25^	Posterior glenoid wear with more than 15 degrees of retroversion	Stepped and monoblock	Polyethylene (Global Steptech)	3 mm (*n* = 20), 5 mm (*n* = 25), and 7 mm (*n* = 4)
Rice et al.^26^	Advanced glenoid humorous with posterior glenoid wear	N/R	N/R	N/R
Favorito et al.^27^	Glenoid osteoarthritis >15° retroversion, and rheumatoid arthritis (*n* = 2)	Stepped and monoblock	Polyethylene (Global Steptech)	N/R
Cil et al.^8^	Osteoarthritis (*n* = 22), post-traumatic arthritis (*n* = 2), rheumatoid arthritis (*n* = 1). The cause of revision in the remaining 13 patients was failed hemiarthroplasty in five patients and aseptic loosening or instability of a total shoulder replacement in 8 patients	N/R	Metal	N/R
Das et al.^29^	Glenohumeral osteoarthritis	Half wedge and monoblock	Polyethylene (Aquaelis)	Posterior augments of 15°, 25° or 35°
Grey et al.^30^	Walch B1 (*N* = 10), Walch B2 (*N* = 46), Walch B3 (*N* = 12)	Full wedge and monoblock	Polyethylene (Equinoxe)	8°
Kohan et al.^28^	Glenohumeral osteoarthritis	Stepped	N/R	N/R

SG: standard glenoid; TSA: total shoulder arthroplasty.

### Type of AG implants used

Only three studies (17%) did not report on the type of glenoid augment used. Fifteen (83%) reported the type and material of the augment. In most studies (*n* = 12, 80%), the implants used were all-polyethylene glenoid augments. Two studies reported AGs from trabecular metal (*n* = 2, 13%) and one a mix between both (*n* = 1, 7%). Out of the 556 augmented a-TSA with identifiable augment material, 81% (452) used all-polyethylene implants, and 19% (104) used metal back augments. The most common design used was a full-wedge all-polyethylene implant (*n* = 248, 45%) followed by a stepped all-polyethylene implant (*n* = 213, 38%) and a half-wedge metal back augmented implant (*n* = 95, 17%).

### Clinical and radiological outcomes

Functional assessment scores were reported in 10 studies (56%; *n* = 374 patients). The American Shoulder and Elbow Surgeons (ASES) score was the most used (Table 3). Ten studies (56%) reported postoperative range of motion (ROM) outcomes. However, functional and ROM outcomes varied significantly between the studies as some reported only postoperative outcomes while others reported both pre- and postoperative outcomes. Of the eight studies that reported baseline and postoperative ROM values, all showed significant improvements after the surgery. All studies showed satisfactory patient-reported outcomes. The six studies reporting baseline functional outcome values improved significantly after the surgery. Further details can be found in Table 3.

**Table 3.** Patient-reported outcome measures (PROMs).

References	Constant score	UCLA score	ASES score	SST	SPADI	VAS
Wright et al.^15^	Pre-Op: N/R Post-Op: 74.9 ± 12.0	Pre-Op: N/R Post-Op: 31.7 ± 4.7	Pre-Op: N/R Post-Op: 90.6 ± 14.0	N/R	Pre-Op: N/R Post-Op: 12.3 ± 18.8	N/R
Michael et al.^16^	*PAG 8° cage*: Pre-Op: 42 Post-Op: 73 *PAG 8° poly*: Pre-Op: 39 Post-Op: 74 *PAG 16° poly*: Pre-Op: 38 Post-Op: 59	N/R	*PAG 8° cage*: Pre-Op:41 Post-Op: 91 *PAG 8° poly*: Pre-Op: 43 Post-Op: 89 *PAG 16° poly*: Pre-Op: 41 Post-Op: 74	N/R	N/R	N/R
Stephens et al.^22^	N/R	N/R	Pre-Op: 39 Post-Op: 91	Pre-Op: 2.4 Post-Op: 10.6	N/R	Pre-Op: 6.3 Post-Op: 0.3
Priddy et al.^24^	*Augmented* Pre-Op: 47.6 ± 15.0 Post-Op: 82.7 ± 12.9 *Control*: Pre-Op: 41.2 ± 13.0 Post-Op: 75.1 ± 13.2 Clinical improvement [augmented vs. control]: 36.0 ± 15.9 vs. 37.1 ± 15.1 (*p* = .8)	*Augmented* Pre-Op: 16.1 ± 3.5 Post-Op: 31.3 ± 4.9 *Control*: Pre-Op: 14.8 ± 3.0 Post-Op: 28.4 ± 5.6 Clinical improvement [augmented vs. control]: 15.2 ± 5.9 vs. 14.7 ± 6.1 (*p* = .7)	*Augmented* Pre-Op: 45.3 ± 12.3 Post-Op: 86.8 ± 14.7 *Control*: Pre-Op: 37.4 ± 12.8 Post-Op: 78.6 ± 18.4 Clinical improvement [augmented vs. control]: 41.5 ± 18.5 vs. 44.6 ± 18.5 (*p* = .5)	N/R	N/R	*Augmented* Pre-Op (Average): 5.0 ± 1.8 Post-Op: 1.1 ± 1.7 *Control (Average)*: Pre-Op: 6.3 ± 2.0 Post-Op: 2.2 ± 2.3 Clinical improvement [augmented vs. control]: 4.0 ± 2.7 vs. 4.4 ± 2.4 (*p* = .4)
Cil et al.^8^	N/R	N/R	Pre-Op: 55 Post-Op: N/R	N/R	N/R	N/R
Grey et al.^30^	Pre-Op: 43.2 ± 13.4 Post-Op: 79.3 ± 10.4	Pre-Op: 15.7 ± 3.8 Post-Op: 32.4 ± 3.6	Pre-Op: 42.2 ± 14.0 Post-Op: 90.3 ± 12.9	Pre-Op: 5.7 ± 2.8 Post-Op: 11.4 ± 1.4	Pre-Op: 77.0 ± 18.8 Post-Op: 10.5 ± 16.4	N/R
Kohan et al.^28^	N/R	N/R	Augmented component 93.3 (*p* = 0.217) Standard component 85.7 (*p* = 0.217)	N/R	N/R	N/R

UCLA: University of California, Los Angeles, ASES: American Shoulder and Elbow Surgeons, SPADI: shoulder pain and disability index, VAS: visual analogue scale, SST: simple shoulder test; PAG: posterior augmented glenoid with anatomic total shoulder arthroplasty.

Data are presented as mean ± standard deviation.

*Note*. The following studies did not report any complications (Cronin et al.,^14^ Sabesan et al.,^17^ Ricchetti et al.,^18^ Lenart et al.,^19^ Terrier et al.,^20^ Sandow and Tu,^21^ Ho et al.,^23^ Ko et al.,^25^ Rice et al.,^26^ Favorito et al.,^27^ and Das et al.^29^).

#### Glenoid loosening

Four studies (28%, *n* = 119) reported radiographic loosening. Grey et al.^30^ report two patients (2.9%) underwent a revision for aseptic glenoid loosening (both consistent with B2 glenoids) approximately 6 months post augmented a-TSA.^30^ Half of the studies (nine) reported various forms of radiological lucency around the glenoid implant ranging from 15% to 58%.

Grey et al.^30^ reported glenoid components implanted in excessive retroversion are associated with increased stress in the implant, bone and cement, all of which increase the risk of aseptic glenoid loosening and the need for revision surgery.^30^

#### Humeral head subluxation

Two studies commented on humeral head subluxation. Stephens et al.^22^ showed correction of humeral head subluxation but declared that studies with longer follow-ups are needed to determine whether the correction and implant fixation can be sustained.^22^ Furthermore, Grey et al.^30^ reported an average humeral head subluxation of 47.4%, with humeral head re-centring on the 8° posteriorly AG implant for all Walch B glenoid types at final follow-up.^30^ No significant differences were observed in the average subluxation percentage between Walch B glenoid types.

### Complications and revision surgeries

Complications and revision surgeries were diverse and not always reported. Twelve studies reported complications (67%). Nine studies (50%, *n* = 431) reported information regarding the need for revision surgeries. From those, four studies reported no revision surgeries in their included participants. Of 800 participants, 17 required revision surgeries. Reasons for revision surgeries included infection (*n* = 4), glenoid/humeral loosing (*n* = 6), anterior/posterior dislocations (*n* = 2), material wear (*n* = 1), fractures (*n* = 1), instability (*n* = 2) and tendon repair (*n* = 1). Further details can be found in Table 4.

**Table 4.** Complications, revision surgeries and radiographic findings.

References	Complications	Revision
Cronin et al.^14^	N/R	N/R
Wright et al.^15^	AG group (*N* = 0) SG group (*N* = 1)	N/R
Michael et al.^16^	Composite cage augment (*N* = 2) 1 Glenoid loosening accident 1 intraoperative avulsion & tuberosity fixation 8° all-polyethylene group (*N* = 0) 16° all-polyethylene group (*N* = 3) 1 cardiac event (unrelated to implant) 2 glenoid loosening	Loosening of glenoid component in 16° all-polyethylene group (*N* = 2)
Sabesan et al.^17^	N/R	N/R
Ricchetti et al.^18^	N/R	N/R
Lenart et al.^19^	0	0
Terrier et al.^20^	0	N/R
Sandow and Tu^21^	Infection (*N* = 1), minor peg perforation (*N* = 1)	Reverse TSA (*N* = 1)
Stephens et al.^22^	0	0
Ho et al.^23^	N/R	0
Priddy et al.^24^	Acute haematogenous infection (*N* = 1) Glenoid component loosening (*N* = 1)	Resection arthroplasty (*N* = 1) Hemiarthroplasty (*N* = 1)
Ko et al.^25^	Perforation (N = 1)	N/R
Rice et al.^26^	N/R	N/R
Favorito et al.^27^	Anterior dislocation (*N* = 1) Posterior dislocation (*N* = 1)	(N = 2) Anterior dislocation patient; after surgery, VAS pain score decreased from 10 to 5, subjective shoulder score was 40 and ASES score was 38 Posterior dislocation patient: underwent reverse TSA secondary to traumatic posterior dislocation 30 months after arthroplasty.
Cil et al.^8^	Proximal humerus fracture: (*N* = 1) Three patients had the removal of components. Two of these operations were due to deep infection at 1.6 and 4.6 months from surgery. One patient had supraspinatus and infraspinatus tendon repair	Reoperation (*N* = 1), proximal humerus fracture (*N* = 1), revision of humeral or glenoid components (*N* = 7) revision surgery secondary to anterior instability (*N* = 2) polyethylene wear with metallic synovitis and severe glenoid osteolysis (*N* = 1)
Das et al.^29^	0	0
Grey et al.^30^	Axillary neurapraxia: (*N* = 2)	N/R
Kohan et al.^28^	*Postoperative complications* Standard glenoid component Pulmonary embolism: 1 Ulnar neuropathy: 1 Superficial infection: 1 Augmented glenoid component Axillary neuropathy: 1 Periprosthetic humeral shaft fx: 1 Superficial infection: 1	0

AG: augmented glenoid; ASES: American Shoulder and Elbow Surgeons; SG: standard glenoid; TSA: total shoulder arthroplasty; VAS: visual analogue scale.

## Discussion

Our review showed that patients who underwent an a-TSA with AG components have had satisfactory short-to-mid-term clinical outcomes. Although some signs of lucency were observed around the components in imaging studies, most patients remained asymptomatic and revision surgery due to loosening was rare up to 5 years. However, caution should be exercised when interpreting these results as there is limited data on patients with long-term follow-up, and the revision rates of the glenoid components could be higher in those patients.

Most patients underwent an a-TSA with AG components of between 5° and 10° secondary to primary glenohumeral osteoarthritis with a B2 glenoid type. In this population, AG components seem to be a reliable and safe alternative. The primary material of the a-TSA was all-polyethylene stepped monoblock AG implants. Unfortunately, our sample size was insufficient to conduct a subanalysis based on the type and material of the augmented components.

One major concern with AG components is the potential for early loosening. Lucency, a radiological sign that could suggest loosening, has been observed around the glenoid component and has been reported both in AG and SG components. Schoch et al.^31^ reported that up to 35% of patients had some degree of radiolucency around the glenoid component. Their study found that peri-implant glenoid radiological lucencies following a-TSA with AG components were associated with lower forward flexion and patient-reported outcomes. However, these differences did not exceed the minimal clinically important difference when glenoid radiological lucencies were below grade 5. Sadly, our review encountered inconsistent and non-standardized reporting of glenoid radiological lucencies, making it difficult to draw firm conclusions.

The incidence of complications and revision surgeries was not reported in many studies included in this review. However, the subgroup of patients who underwent an a-TSA with larger augments (≥15°) had a higher rate of revision surgery (4 out of 44, 9%). Revisions due to aseptic loosening were seen only in this group only. Nevertheless, larger augments are used for more severe deformities which may introduce a selection bias. Although this was found in a small subgroup, caution is warranted when considering larger augments. Despite this, a-TSA with AG components can still be a good alternative to manage common patterns of glenoid wear associated with osteoarthritis, avoiding some of the pitfalls of asymmetric reaming and RSA.^28^

This systematic review provides insight into the clinical outcomes and safety of a-TSA with AG components, but controversies persist. There is a lack of comparison with other surgical options such as eccentric reaming with standard components, bone grafting, or RSA. It is unclear which glenoid wear patterns benefit from each option, and patient factors that impact outcomes, as well as cut-off values of glenoid dysplasia for using augmented components, are still up for debate.

### Limitations

The present review has limitations. First, the lack of any prospective randomized clinical trials. Thus, the non-random samples could imbalance certain patient or procedure factors. Also, the total sample size of this cohort is still limited. Therefore, this review's findings should be interpreted with caution as they are based on low-to-moderate quality evidence. However, the information provided is valuable in understanding the outcomes of a-TSA with AG in what is expected to be a growing patient population. Further research is needed to better understand the role and long-term safety profile of this treatment and to compare it to other methods for addressing glenoid wear.

## Conclusion

Our review suggests that a-TSA with AG components can lead to similar mid-term outcomes as those seen with SG components. However, it remains uncertain if these results will persist in the long term due to high rates of glenoid component lucency during follow-up. Further research with longer follow-up periods and data from national surgical registries that provide larger sample sizes is necessary to fully understand the safety and effectiveness of a-TSA with AG components.

## Supplemental Material

sj-docx-1-sel-10.1177_17585732231192991 - Supplemental material for Outcomes of patients undergoing anatomical total shoulder arthroplasty with augmented glenoid components – a systematic review Supplemental material, sj-docx-1-sel-10.1177_17585732231192991 for Outcomes of patients undergoing anatomical total shoulder arthroplasty with augmented glenoid components – a systematic review by Carlos Prada, Omar A Al-Mohrej, Salwa Siddiqui, and Moin Khan in Shoulder & Elbow
