# Supplementary material for: Outcomes of patients undergoing anatomical total shoulder arthroplasty with augmented glenoid components – a systematic review
Source: Shoulder Elbow. 2023 Aug 7;16(5):462–73. doi: 10.1177/17585732231192991 (PMC11523180; doi:10.1177/17585732231192991)
Supplement: sj-docx-1-sel-10.1177_17585732231192991 - Supplemental material for Outcomes of patients undergoing anatomical total shoulder arthroplasty with augmented glenoid components – a systematic review [file sj-docx-1-sel-10.1177_17585732231192991.docx]

**Appendix 1:** Search Strategy

| **Database** | **Search Fields** | **Results** |
| --- | --- | --- |
| CINHAL | 1. Arthroplasty, Replacement, Shoulder+ | 480 |
| Medline | 1. exp Arthroplasty, Replacement, Shoulder/ and shoulder arthroplasty.mp. | 1208 |
|  | 1. augm*.mp. | 192077 |
|  | 1. 2 and 3 | 49 |
| Embase | 1. exp shoulder arthroplasty/ | 5129 |
|  | 1. augm*.mp. | 249538 |
|  | 1. 5 and 6 | 208 |
| Pubmed | 1. (("augment"[All Fields] OR "augmentation"[All Fields] OR "augmentations"[All Fields] OR "augmented"[All Fields] OR "augmenting"[All Fields] OR "augments"[All Fields]) AND ("shoulder"[MeSH Terms] OR "shoulder"[All Fields] OR "shoulders"[All Fields] OR "shoulder s"[All Fields]) AND ("arthroplasty"[MeSH Terms] OR "arthroplasty"[All Fields] OR "arthroplasties"[All Fields])) AND (fha[Filter]) | 252 |
